# The Complicated Relationship between Dieting, Dietary Restraint, Caloric Restriction, and Eating Disorders: Is a Shift in Public Health Messaging Warranted?

**DOI:** 10.3390/ijerph19010491

**Published:** 2022-01-03

**Authors:** Tiffany M. Stewart, Corby K. Martin, Donald A. Williamson

**Affiliations:** Pennington Biomedical Research Center, Baton Rouge, LA 70808, USA; Martin@pbrc.edu (C.K.M.); Donald.Williamson@pbrc.edu (D.A.W.)

**Keywords:** dietary restraint, dieting, eating disorders, disordered eating, bulimia nervosa, anorexia nervosa, caloric restriction, obesity, weight loss, binge eating

## Abstract

The origins of theories specifying dietary restraint as a cause of eating disorders can be traced to the 1970s. This paper will present an overview of the origins of dietary restraint theories and a brief historical review of evidence will be summarized. Recent research will be presented, including the results from the CALERIE Phase 1 study, as well as CALERIE Phase 2, which were NIH-sponsored randomized controlled trials. CALERIE 2 provided a test of the effect of two years of caloric restriction (CR) on the development of eating disorder syndromes and symptoms in comparison to a control group that did not alter eating behavior or body weight. The intervention was effective for inducing a chronic (two-year) reduction in total energy expenditure and increased dietary restraint but did not increase symptoms of eating disorders. The results of this recent investigation and other studies have not provided experimental support for conventional dietary restraint theories of eating disorders. These findings are discussed in terms of potential revisions of dietary restraint theory, as well as the implications for a paradigm shift in public health messaging related to dieting.

## 1. Origin and Evolution of Dietary Restraint Theories: From Past to Present

The hypothesis that dieting, dietary restraint, and caloric restriction cause harmful overeating, weight gain, obesity, and eating disorders was initially proposed in a paper that described the results of a laboratory study of eating behavior [1]. Although used somewhat interchangeably, the terms dieting, dietary restraint, and caloric restriction have differences in meaning and in connotations. For purposes of clarity, the following definitions will be used to define these three terms throughout this paper. The term dieting has been used as a catch all descriptor for restrictive eating of any kind. However, dieting has been defined in the literature as adherence of a specific eating plan with the goal of either losing or maintaining weight [2,3]. It is important to consider that in the case of dieting, adherence to an eating plan with the goal of losing weight could include fad diets, do-it-yourself diets, or diets of proven efficacy deployed under the care of a qualified professional. Dietary restraint refers specifically to the cognitive effort to restrict food intake, or the intent to control food intake [4]. Caloric restriction (CR) refers to the actual reduction in calories consumed to create and energy deficit, regardless of intention [2]. The nuance pertaining to intention may be fundamental to understanding the implications of these constructs. All three terms require the same goal (to manage calorie intake), but only dieting and CR include behavioral actions, e.g., eating less food, as dietary restraint is a cognitive construct that may or may not be associated with restricting energy intake. As noted in the next paragraph, much of the research has focused on the induction of dietary restraint, not actual reductions in food or energy intake. Thus, most studies examining the relationship between dietary restraint and eating disorders have not employed measurement of an actual reduction in calorie intake, but mainly measures of the intent to reduce intake, via self-report questionnaires.

The notion that the intent to control food intake (dietary restraint) resulted in counter-regulatory eating behavior in response to violation of personal eating norms was formulated by modifying an earlier set of theories that hypothesized that obesity and overeating were caused by either: (1) failure to regulate eating on the basis of an internal physiological state, e.g., feelings of anxiety were confused as being feelings of hunger [5], called the psychosomatic hypothesis, or (2) people with obesity were unusually responsive to external cues [6], which was called the externality hypothesis.

By 1985, the intent to restrict food intake to manage weight gain was deemed to be a “cause” of binge eating [7]. These concerns were linked to the seminal findings of the Keys [8] study of severe fasting in 32 conscientious objectors who were healthy young men. Keys found that restrictive eating resulted in altered perceptions and behaviors around food, including obsession with and possessiveness over food. The men were placed on a dietary regimen that included a 12 week control period and then a 24 week semi-starvation period. During the semi-starvation period, caloric intake was established with the goal of an average weight loss of 24 percent. Following the semi-starvation period, a rehabilitation phase occurred, during which participants suffered from inadequate hunger cues and engaged in binge eating and purging.

Concerns were also linked to reports that repeated dieting followed by regaining weight (called weight cycling or yo-yo dieting) could have harmful metabolic and behavioral effects [9]. The hypothesis originated by Herman and Polivy [1] that dietary restraint can lead to the development of eating disorders was later labeled as the dietary restraint theory by Williamson [10] and has formed the basis of concerns about harms caused by dieting, CR, and weight management [2]. By the time this theory came into focus, dietary restraint was implicated in the etiology of anorexia nervosa, bulimia nervosa, and compulsive overeating [10,11,12].

During the early 2000s, papers [13] and books [14] provided cautionary warnings about the potential harms that might be derived from dietary restriction and weight management. All the while an epidemic of obesity was unfolding, with safe and effective lifestyle modification interventions being tested as one solution to the problem of excess adiposity [15,16]. Simultaneously, suggestions were emerging that chronic caloric restriction might be helpful to promote healthy aging [17,18]; however, concerns were also expressed about employing CR in healthy overweight people [19] due the potential for triggering overeating, binge eating, or eating disorders. The net result of these theories and research was that serious concerns about the safety of any dieting were raised [2], public health warnings were presented [20], and confusion about the safety and efficacy of weight management became commonplace [14,21]. Discussion [22] and research [23] about the potential causal relationship between dietary restraint and overeating have continued to the present time period.

## 2. Early Evidence concerning Dietary Restraint Theories: 1975–1990

The earliest studies of the hypothesis that intent to restrict food intake or diet could result in unintentional overeating were conducted in controlled eating laboratories [1,24]. These studies reported that in response to eating a calorically dense pre-load, dieters (but not non-dieters) tended to overeat in a second eating episode, which was termed “counter-regulatory eating”. Numerous cross-sectional studies reported significant correlations between self-report measures of dietary restraint and overeating or symptoms of eating disorders [25,26,27]. Thus, by 1990, dietary restraint was viewed as a well-established risk factor for the development of excessive eating, obesity, and eating disorders.

## 3. Later Evidence concerning Dietary Restraint Theories: 1990–2021

During the late 1990s, Stice and colleagues [28,29] hypothesized and tested a dual-pathway model of bulimia nervosa that posited the dual influences of negative affect and restrained eating on the development of eating disorder symptoms. These “dual factors” were hypothesized to be moderated by sociocultural pressure for thinness and ideal body internalization that determined level of body dissatisfaction. This theory hypothesizes that sociocultural pressure for obtaining the thin beauty ideal increases the risk for body dissatisfaction, which increases the risk for negative affect and dietary restriction, which then increases the risk of the onset of eating disorder symptoms, e.g., binge eating. This model was supported in other research by Goldschmidt and colleagues [30,31]. Furthermore, other longitudinal studies by Stice and colleagues [32] suggested that successful dietary restraint (actual self-regulation of food intake) might result in reduced binge eating.

Lowe [33] also made an effort to address the conflicting findings associated with dietary restraint theory in the early 1990s, proposing a three-factor model of dieting. This model was used to reinterpret earlier findings related to restraint theory by layering relationships between the frequency of dieting and overeating, current dieting, and weight suppression, while incorporating possible mediating mechanisms. In this model, restraint is shown to have different effects than current dieting and weight suppression, as restraint was shown to be related to past behavior rather than current state.

More recent cross-sectional studies of dietary restraint theories have been conducted with sophisticated statistical methodology [34] and different conceptualizations of restrictive versus unrestricted eating [35], continuing to report correlations between dietary restraint and overeating, sometimes concurrently with other putative risk factors, e.g., body dissatisfaction. Some longitudinal studies have reported that dieting typically precedes the development of eating disorders [36,37,38]. Other longitudinal studies have suggested that perhaps the relationship between restrained eating and overeating or eating disorders was more complex, e.g., caused by current dieting [39], severe fasting [40], stress [41], or negative affect [42]. Thus, by 2021, numerous prospective risk factor studies had failed to support the primary hypotheses of dietary restraint theories: that it caused binge eating or other eating disorders.

## 4. Manipulation of Dieting, Dietary Restraint, and Caloric Restriction in RCTs

In a critique of risk factor research pertaining to health, Kraemer and colleagues [43] noted that to be termed a “modifiable causal risk factor”, variables (not fixed characteristics) must precede the onset of a health condition, e.g., eating disorders or obesity. Variables that are simply associated with health conditions, e.g., in cross-sectional studies or at baseline observations, can be viewed as correlates of the health condition. Based on early research, it was unclear whether the putative risk factor, dietary restraint, caused binge eating and eating disorder symptoms or vice versa. Kraemer and colleagues [43] also observed that the best test of whether a putative risk factor plays a causal role in the development of a health condition is to test the manipulation of the putative risk factor in a controlled experiment, e.g., a randomized controlled trial (RCT), to observe whether the health condition is impacted in the experimental arm, relative to the control arm.

Starting in the 1990s, a series of RCTs tested whether the manipulation of intentional dieting resulted in increased overeating, binge eating, and/or eating disorder symptoms [44,45,46,47,48] in diverse cohorts ranging in age, sex, and weight status. Some of these studies reported that intentional food restriction not only did not result in excessive eating, but it resulted in actual improvement of overeating, binge eating, and excess adiposity. However, there were two primary limitations of these RCTs: (1) objective measurements of chronic energy imbalance caused by the behavioral interventions were not obtained and (2) in most studies, the cohorts included participants who were overweight or had obesity. This second limitation is based on the common observation that eating disorders, such as anorexia nervosa and bulimia nervosa, are most often observed in normal-weight young adults and adolescent girls. Objective measurements of caloric intake are important to determine energy imbalance without user bias and/or error. Further, although some studies attempted to measure caloric intake through self-report food diaries [45,46], many of these measures captured variables other than calorie intake such as self-report restrained eating and binge eating scales [47,48].

In response to these limitations, Williamson and colleagues [49] reported the results of a six-month pilot study called the Comprehensive Assessment of the Long-term Effects of Reducing Energy Intake (CALERIE, Phase 1 at Pennington Biomedical Research Center) that compared three different approaches for inducing long-term caloric restriction to a control group that did not significantly modify food intake. The primary aim(s) of the CALERIE Phase 1 study was to test different strategies for inducing long-term caloric restriction in humans who were overweight, but did not have obesity (body mass index (BMI) range was 25–30 kg/m^2^); and to test the effects of caloric restriction on biomarkers for longevity. The study found evidence that sustained caloric restriction modified some biomarkers of aging and metabolism [50]. The study carefully monitored changes in energy balance using objective measures (doubly-labeled water and changes in body composition) and also observed changes in body weight (and composition). Changes in measures of dietary restraint, overeating, and eating disorder symptoms were also monitored using a validated self-report inventory called the Multidimensional Assessment of Eating Disorder Symptoms (MAEDS) [51]. Of particular relevance to this discussion were two arms of the RCT (in comparison to the control arm): (1) caloric restriction (CR, prescribed average 25% reduction in caloric intake) and (2) low-calorie diet (LCD, also prescribed average 25% reduction in caloric intake). The pilot study found that the average reduction in caloric intake for the CR arm (relative to the control arm) was ~13% and for the LCD arm was ~19%. The study found that CR and LCD (relative to the control arm) increased self-reported dietary restraint, but binge eating was reduced and other eating disorder symptoms did not worsen. Of interest was the observation that avoidance of “forbidden foods”, i.e., foods with high dietary fat or added sugar, increased in both arms, which is consistent with the lifestyle change, healthy eating instructions for the two interventions. Additionally, concerns about body size, as measured by the Body Shape Questionnaire (BSQ) [52] were reduced in both CR and LCD, which might be expected since the average weight loss for the CR arm was ~10% and for the LCD arm was ~13%.

Another paper from this study compared the validity of different self-report measures of dietary restraint as a measure of intent to diet and actual calorie reduction [53]. The study found that the Restraint Scale of the Eating Inventory (EI) [54] was most valid for measuring intent to restrict and changes in the EI Restraint Scale were most valid for measuring caloric restriction, i.e., actual dietary restriction. We will return to this discussion in the next sections that describes the results of CALERIE Phase 2 and types of dietary restraint. The primary findings of the Williamson et al. [49] pilot study were that in adults who were overweight, but did not have obesity, significant long-term energy imbalance, using objective measures, resulted in significantly increased dietary restraint and weight loss, but reduced self-reported binge eating, lowered concerns about body size and shape, and did not result in the development of eating disorder symptoms.

When combined with the findings of other RCTs and longitudinal studies from 1990 to 2015, the results from CALERIE pilot study led to calls for re-evaluation of dietary restraint theories of eating disorders [2,55,56]. One limitation in this RCT was following participants up only to six months. Although six-month studies are adequate tests of the original theory of dietary restraint leading to counter regulation, naturalistic long-term cases of ED development may represent a different scenario that may surface past the six-month mark.

The CALERIE Phase 2 trial was designed in response to the findings of the Phase 1 studies [50,57,58] with the intent to test a longer (two-year) period of caloric restriction in adults with slightly lower BMI range (22–28 kg/m^2^) in comparison to CALERIE Phase 1 studies. The research design [59] required randomization to one of two arms (CR-25% or ad lib control) in a 2:1 ratio. The CR intervention used the methodology of recent intensive lifestyle interventions (ILIs) [60] to promote adherence to a healthy diet that was 25% calorically restricted. The primary aims of the phase 2 study were similar to those of the initial pilot studies and careful biological, psychological, and behavioral measures were obtained at approximately six-month intervals. The total number of participants across three sites was 218 adults between the ages of 21 to 51 years. Participants were carefully screened prior to enrollment and randomization [61]. Of relevance to this paper, participants were screened for the presence of behavioral or psychiatric problems, which included but were not limited to full syndrome or significant symptoms (subclinical cases) of substance use/abuse, depression, anxiety, eating disorders, and/or pharmacologic treatment for a psychiatric disorder. If psychiatric disorders, subclinical eating disorders, or significant body image disturbance were identified, participants were excluded from participation in the study. Out of 1069 people who attended the full in-clinic screening (which included psychiatric disorders), approximately 2% at each site were excluded for the presence of any significant psychiatric issue. The small number of potential patients presenting excludable conditions may be the result of a skewed voluntary sample. For example, patients without psychiatric disorders may be more adherent to dietary restriction and may have more realistic goals compared to those screened out for psychiatric symptoms [62]. Further, excluding psychiatric disorders such as anxiety was important to screen out those at higher risk from developing an eating disorder from caloric restriction, as anxiety often precedes subclinical and full eating disorder diagnoses. The findings of the study that are described for this paper were designed to follow-up the results of the Williamson et al. [49] study and were a critical part of the safety protocol for the primary study.

The CALERIE Phase 2 study resulted in significant weight loss (~10%) in the CR arm, with average reductions in energy balance of ~12% over the two years. Caloric restriction was greatest during the first six months of the study and then gradually relaxed over the next 18 months [63]. Evidence for improved age-related outcomes was found. As described by Dorling et al. [64,65], dietary restraint, measured by the EI Restraint Scale, increased significantly in the CR arm, but not in the control arm. However, increased binge eating and eating disorder symptoms were not observed as predicted by dietary restraint theories [65]. Additionally, similar to the findings from CALERIE Phase 1 trial, avoidance of “forbidden foods” was increased by participation in the CR intervention. As part of the safety protocol, markers of eating disorders were monitored using the MAEDS and if symptoms of an eating disorder were observed, participants were interviewed for a diagnosis of an eating disorder using the Interview for Diagnosis of Eating Disorders [66]. Of central importance to the primary hypothesis of dietary restraint theories, no eating disorders (in either arm) were identified during the two-year RCT [65,67]. A reduction in binge eating, measured using the MAEDS, was observed in the CR arm and reductions in concerns about body size and shape, measured by the BSQ, were also found for the CR arm. No other symptoms of eating disorders, including changes in food cravings were observed. In general, the CR intervention was associated with few physical/medical adverse events [67] and overall improved psychological well-being and quality of life [65,68].

In summary, the CALERIE Phase 2 study was a critical test of the theory that manipulation of dietary restraint (by inducing CR in one arm but not the control arm) would result in increased binge eating, overeating, or development of eating disorders or eating disorder symptoms in the CR arm but not the control arm. The study included approximately 50% normal-weight and 50% slightly overweight adults over two years of prolonged CR. No evidence supporting the predictions of dietary restraint theories was reported.

## 5. Implications of Findings Past and Present

### 5.1. The Definition of Dietary Restraint

Over the past 50 years, many questions have been raised about the definition of dietary restraint and whether the original definition of the term, as measured by the Restraint Scale [1], satisfactorily addresses these questions. One of the first authors to raise this question was Westenhoefer [69] who reported two types of dietary restraint: (1) rigid dieting that was associated with eating disorder symptoms and (2) flexible dieting that was associated with the absence of eating disorder symptoms. Studies published over the next 15 years [70,71,72] replicated these findings.

### 5.2. The Measurement of Dietary Restraint

Many measures of the dietary restraint construct have been developed [53] and questions about the validity of these measures have been raised over time [73]. For example, studies have found that individuals with elevated dietary restraint scores are at an increased risk for the development of bulimic symptoms. However, studies have also found that participation in structured, professional-led interventions to reduce food intake have reduced symptoms of bulimia nervosa. As noted earlier, these contradictory findings indicate that the dietary restraint measures from trials that showed increased risk of eating disorder development were not effectively identifying individuals that were actually reducing food intake [73]. Further, a recent study [74] re-examined the factor structure of different questions from five self-report inventories pertaining to dietary restraint: (1) the Dutch Eating Behavior Questionnaire (DEBQ) [75], (2) the Eating Disorder Inventory 3 (EDI-3) [76], (3) the Eating Disorder Examination-Questionnaire (EDE-Q) [77], (4) the Restraint Scale (RS) [1], and (5) the EI Restraint Scale (EI-R) [78]. The psychometric study found that the questions from different measures loaded on three factors and did not represent a unitary construct of dietary restraint. The three factors were labeled: (1) calorie counting, (2) preoccupation with dieting, and (3) weight-focused restraint. Additionally, questions have been raised [79,80,81] as to whether all dietary restraint scales measure intent to diet versus actual dietary restriction. As noted earlier, Williamson et al. [53] concluded that the EI Restraint Scale was most valid for measuring intent to diet and that changes in the EI Restraint Scale were most valid for measuring actual dietary restriction. These findings suggest that the very significant increases in the EI Restraint Scale scores observed in the two CALERIE studies (Phases 1 and 2) represent strong tests that increased dietary restraint but did not result in the development of eating disorders.

### 5.3. Dietary Restraint a Risk Factor OR a Correlate of Eating Disorder Symptoms

Longitudinal data do support dietary restriction as a risk factor, but the literature overall is somewhat mixed [28]. As noted by Kraemer et al. [43], it is quite possible that some putative risk factors for health conditions are simply correlates of the health conditions, meaning that the direction of causation is not specified by cross-sectional or longitudinal studies that rely upon correlation statistics. Indeed, some studies [29,82] have reported evidence that increased dietary restraint and/or restricted eating is often a result of binge eating and should not be conceptualized as a cause of binge eating. Given current evidence from RCTs, this possible explanation for earlier findings concerning the association of dietary restraint and overeating or binge eating may be viable.

### 5.4. Susceptibility of Adolescents or Young Adults to the Effects of Dietary Restraint: Obesity and Eating Disorder Prevention and Intervention

Most of the RCTs pertaining to increased dietary restraint as a cause of eating disorders have studied adults. Nonetheless, eating disorders (especially anorexia and bulimia nervosa) are most often observed in younger people during adolescence or young adulthood. As the obesity epidemic has worsened, it has become imperative to find prevention and intervention efforts that are safe and effective for young people. One questionnaire study evaluating the relationship of body image and cognitive restraint to obesity found marginal associations through correlational analyses [83]. Related to this, it is important to recognize the association between self-reported measures of body dissatisfaction and obesity (e.g., increased BMI). Often, when it is reported that increased dietary restraint is correlated with body dissatisfaction when body dissatisfaction is correlated with higher BMI, body dissatisfaction is described as the driver of dietary restraint. In fact, dietary restraint and increased BMI are the primary factors in this relationship and body dissatisfaction may play an exacerbating role. The use of self-report questionnaires to measure body dissatisfaction may not allow an adequate test of this complex relationship. In essence, it can be hypothesized that obesity is associated with body dissatisfaction and this drives dietary restraint. When dietary restraint is successful and weight loss occurs, body dissatisfaction goes down, as this has been shown empirically in clinical trials [65]. Thus, the causal role of dietary restraint in the development of body dissatisfaction and obesity is in question.

Many other obesity prevention studies in children and adolescents have utilized RCT methodology and have investigated whether weight gain prevention interventions had the effect of increasing eating disorder symptoms. Overall, these studies have either reported no significant harm (by leading to the development of eating disorders) or a reduction in eating disorder symptoms [56]. Given that obesity and disordered eating behaviors have shared risk factors [84], it makes sense to combine obesity and eating disorder prevention efforts. Programs such as this, aimed at healthy weight management with the goal of preventing obesity and disordered eating [85], that have incorporated the promotion of healthy eating and exercise have had no significant adverse effects, but rather beneficial effects of reducing eating disorder symptoms [32,40,86,87,88]. Of note, rather than a strict focus on calorie reduction, these interventions provided content on topics such as food being fuel for the body and making healthier food swaps from less nutritious foods to more nutritious foods. Recent studies of obesity and ED treatment interventions in adolescents [89,90] have concluded that structured obesity treatment programs for adolescents reduce eating disorder prevalence, risk, and symptoms, [90] as well as reducing shape and weight concerns [91]. These findings also suggest that while these “supervised” interventions are not associated with increased eating pathology, unsupervised “dieting”, as a self-reported method to lose weight, e.g., fasting, skipping meals, and diet pill use, has been associated with increased eating pathology among adolescents [92]. Additionally, not all individuals at increased risk maybe adequately be identified in aggregate data [93]. These findings imply that, with proper screening and continued monitoring of eating disorder risk factors and related behaviors, concerns about eating disorder development are not valid reasons to withhold delivery of obesity prevention or weight management interventions to adolescents and young adults [92]. Taken together, these findings suggest that structured, multi-component, evidence-based obesity and eating disorder prevention programs do not increase risk for the development of eating disorders.

### 5.5. Interaction of Dietary Restraint and Vulnerability to Eating Disorders

Based on current evidence, it is now clear that dietary restraint alone does not cause the development of eating disorder symptoms. It is plausible that dietary restraint may activate putative vulnerability factors that result in eating disorder symptoms in people who are vulnerable to this development. One possibility is that the etiology of eating disorders is best understood to be caused by a gene–environment interaction, with dietary restraint possibly functioning as an environmental trigger, though dietary restraint could also be simply a correlate of eating disorder symptoms. Several authors [53,94] have hypothesized that binge eating may be a behavioral phenotype representing a genetic or acquired vulnerability that could be activated by factors such as restricted eating. Taxometric studies of eating disorder symptoms [71,95] have reported evidence that binge eating is best conceptualized as a unique categorical characteristic of eating disorders that fits well with the notion that binge eating may be a phenotype that is qualitatively different from normative eating. Earlier papers speculated that bulimia might share etiology with affective disorders [96,97] or obsessive-compulsive disorder [98], psychiatric syndromes that are often viewed as determined by biological vulnerabilities. As noted earlier, another vulnerability hypothesis of the etiology of bulimia nervosa comes from Stice’s dual-pathway model of bulimia nervosa, which includes interactions of several vulnerability factors including negative affect, dietary restraint, sociocultural pressure for thinness, and idealization of a thin body shape [99]. This model has also been extended to the symptoms of anorexia nervosa [100]. In sum, a vulnerability hypothesis for the development of eating disorders in some, but not all, people has been favored by some experts for over 40 years. Evidence supporting this hypothesis has been mounting for some time.

### 5.6. Dietary Restraint as Healthy Self-Regulation

In a recent review of the dietary restraint theories as they pertain to the development of eating disorders and weight management, Shaumberg and colleagues [2] proposed a model of self-regulation that included dietary restraint as a component of either healthy or unhealthy self-regulation of eating and weight management. In this model, dietary restraint is viewed as a self-regulation strategy and includes the process of curbing the tendency to “eat at will”. According to this proposal, self- regulation includes consistent self-monitoring, realistic goals, accurate self-evaluation, and interruption of behavioral inertia (to eat at will), which can result in healthy weight management, but the antithesis of these strategies (lack of consistent self-monitoring, setting unrealistic goals) can result in the development of disordered eating.

### 5.7. Dietary Restraint and Weight Management

Contrary to the public health concerns of messages derived from dietary restraint theories, recent evidence leads to the conclusion that intention to restrict food intake and actual restriction of dietary intake (CR) are both safe and effective for weight management and promotion of good physical and mental health in those without significant risks for the development of eating disorders. Recently published articles have further supported the emerging consensus that dietary restraint may be practiced in a healthy manner [101]. These conclusions are especially warranted if ILI methodology to promote healthy lifestyle behavior change is utilized. Prospective studies suggest that dietary restraint may be helpful for mitigation of weight gain, but not as a predictor of weight gain [33]. It is relevant to note that although supervised ILI interventions successfully promote weight loss over the short term, long-term weight control is not guaranteed, though the importance of mitigation of weight gain over the long term cannot be ignored. Additionally, weight-loss interventions that promote healthy weight loss but not negative attitudes toward one’s body might result in both weight loss and reductions in eating disorder symptoms and presumably risk of developing eating disorders. This conclusion would be consistent with most modern recommendations about the promotion of healthy eating (e.g., nutrient-rich diet and regular eating) with low concerns about the development of eating disorders [55].

## 6. Conclusions

Results of controlled trials of ILIs for weight management in adults, weight gain prevention, and eating disorder prevention in young people have failed to support the hypotheses of harmful effects caused by intent to diet or actual caloric restriction. Thus, neither the intention to diet nor actual CR appears to reliably result in ED development. Rather, the results of these studies have consistently shown that healthy eating, exercise, and appropriate energy balance behaviors, without the promotion of negative body image, shaming or stigma, can be safe and effective for the enhancement of physical and mental health. Current evidence suggests that dietary restraint is associated with eating disorder symptoms, but it is not necessarily a causal factor in the etiology of eating disorders. In fact, increased dietary restraint may be a consequence of weight gain and self-perceived overeating. One limitation of the studies from which many of these conclusions were obtain, however, is that they relied on supervised interventions from rigorously controlled trials. This is necessary to detect causality, though it highlights that additional research is needed that employs pragmatic and effectiveness designs to determine if negative effects of dieting occur more commonly when structured interventions are deployed on a larger scale and there is less supervision.

Taken collectively, these findings suggest that revision of dietary restraint theories of eating disorders is warranted. These revisions may require additional research about vulnerabilities that make some people more susceptible for the development of eating disorders during certain diets. It is possible that interactions exist, where some specific forms of dieting or intent to diet are more harmful than other forms but only for certain people. Unfortunately, the relationship between different patterns of dietary restraint and harmful effects is currently unknown. That said, avoidance of extreme weight control behavior has been found to be associated with better weight maintenance [102]. Additionally, it is possible that extreme forms of dietary restriction, e.g., chronic fasting, especially in young people, may be more harmful than the types of dietary restraint and evidence-based ILI-guided caloric reduction that have been studied in recent years. However, this possibility has been questioned by findings of the relative safety and beneficial effects of intermittent fasting [103]. That said, at this time, intermittent fasting is not recommended for children or adolescents, the elderly, underweight individuals, pregnant or lactating women, or individuals vulnerable to eating disorders [103].

Over the past 50 years, the term “dieting” has earned a bad reputation in some quarters and a good reputation in others. As aforementioned, unsupervised dieting in adolescents [89], do-it-yourself dieting approaches, promotion of fad diets or methods promoted in the popular media and most recently encouraged through social media platforms may do harm. We realize that this type of diet promotion is pervasive. On the other hand, continuing a dialogue that all forms of dietary restriction are potentially harmful and ineffective for all individuals is not true nor is it useful for public health. We recommend that it is time to leave the term “dieting” to history and get more specific about the language we use and specific recommendations for healthy living. We also recommend that this more unified and consistent messaging should begin with researchers and academics whose work helps form the messaging. Public health messaging must acknowledge the complex relationship between weight and health, including both the risks and benefits of dietary restriction. Thus, more balanced messaging approaches should incorporate evidence-based guidance. Some general examples include (1) discouraging extreme, unsustainable forms of dietary restriction (including media-endorsed fad diets or non-evidence-informed, do-it-yourself strategies; (2) limiting a reduction in caloric intake to guided ILI/evidence-based programming; and (3) improving health behaviors (e.g., nutritious foods and moderate exercise), rather than a limited, specific focus on the outcome of weight loss alone. An example of this type of messaging might include discussion of the functional benefits, e.g., reduced stress and improved mood, of engaging in healthy forms and amounts of exercise instead of marketing health behaviors as weight loss behaviors

Further, public health messaging can be more successful when aligned with community influencers that are already embedded in groups as trustworthy messengers and are known and respected by community members. Finally, issues of equity and access to healthy and affordable foods and services need to be considered for society to more equally benefit from the science and the messaging. Access to messaging is often inequitable due to gaps in technology infrastructure and a lack of diversity in communication approaches [104]. Those with low socioeconomic positions often have less access to health communication [105], supporting the idea that those who are most in need often receive less when it comes to advancements in the health field [106].

In conclusion, the risks and benefits of dieting, dietary restraint, and CR have sparked great debate over many years. In order to maximally benefit public health, we must find the middle ground between the prevention and treatment of obesity and eating disorder prevention [107]. Available scientific evidence points to a conclusion that intentional dietary restriction, when conducted according to empirically validated programs, is both safe and effective for weight management and the prevention of eating disorders in individuals that have not been assessed to be vulnerable or at risk. Thus, it is time to consider a paradigm shift in our health messaging by not only working to eradicate unhealthy and harmful messages encouraging extreme and/or unhealthy dieting practices across platforms such as social media, but also replace overarching, generalized public health messaging about the “dangers of dieting” with messaging that is more nuanced, considering the positive effects of caloric reduction along with messaging about the vulnerability factors that might put some people at risk for the development of eating disorders. Critically, this messaging can simultaneously and directly address anti-fat bias and attitudes and reinforce healthy goals and behaviors for weight management vs. endorsing culturally idealized perceptions related to thinness.

## Data Availability

Data are available online at https://calerie.duke.edu/ (accessed on 26 October 2021).
